# Developing the Digital Health Communication Maturity Model: Systematic Review

**DOI:** 10.2196/68344

**Published:** 2025-04-14

**Authors:** Grace Jeonghyun Kim, Kang Namkoong

**Affiliations:** 1 Department of Communication University of Maryland, College Park College Park, MD United States

**Keywords:** digital health, maturity model, integrated model, digital health communication, health communication, systematic review, model development, health care innovation, digital transformation, organizational readiness, evaluation metrics, health care technology, digital strategy

## Abstract

**Background:**

Digital health has become integral to public health care, advancing how services are accessed, delivered, and managed. Health organizations increasingly assess their digital health maturity to leverage these innovations fully. However, existing digital health maturity models (DHMMs) primarily focus on technology and infrastructure, often neglecting critical communication components.

**Objective:**

This systematic review addresses gaps in DHMMs by identifying deficiencies in user communication elements and proposing the digital health communication maturity model (DHCMM). The DHCMM integrates critical health communication dimensions such as satisfaction, engagement, personalization, and customization to provide a comprehensive evaluation framework.

**Methods:**

We followed the PRISMA (Preferred Reporting Items for Systematic Reviews and Meta-Analyses) guidelines to conduct a systematic review of studies selected from 3 databases: EBSCO, PubMed, and ProQuest. Studies were screened and included based on their focus on digital health maturity and communication elements, with the final selection limited to English-language research addressing DHMMs.

**Results:**

Of the 1138 initially identified studies, 31 (2.72%) met the inclusion criteria. Current DHMMs heavily emphasize infrastructure while overlooking user engagement and communication; for instance, only 35% (11/31) of the reviewed models incorporated user satisfaction, and less than one-fifth (6/31, 19%) addressed personalization or customization. The DHCMM addresses these gaps with 7 maturity levels, ranging from *initial* to *engaged*, and emphasizes user-centered metrics and governance. Quantitative analysis showed substantial variations in communication metrics, with satisfaction metrics incorporated at an average rate of 22% (7/31) across the reviewed models.

**Conclusions:**

The DHCMM shifts the focus of digital health maturity assessments by emphasizing communication and user engagement. This model provides health care organizations with a structured framework to enhance digital health initiatives, leading to better patient outcomes and system-wide efficiencies. The model delivers actionable insights for organizations aiming to achieve advanced digital maturity by addressing underrepresented dimensions. Future research should implement and refine the DHCMM across diverse health care contexts to enhance its effectiveness. The adoption of this model could result in more equitable, user-centered health care systems that integrate technological advancements with human-centered care.

## Introduction

### Background

Advancements in digital technology have ushered in an era characterized by rapid digital transformations across various sectors. This progress has led to the emergence of digital health as a pivotal force in the public health arena, fundamentally reshaping the accessibility, delivery, and management of health care services [1]. Digital media technology enables patient monitoring and facilitates communication between health care providers and patients, representing a revolutionary shift that transcends geographic and temporal limitations [2]. Digital health, also known as eHealth or health IT, holds immense potential to improve patient outcomes, streamline health care processes, and redefine the physician-patient relationship [3].

Digital health encompasses various applications, including mobile health, wearable devices, telemedicine platforms, and electronic health records [4]. These technological advancements enable real-time data exchange, remote patient monitoring, personalized treatment plans, and evidence-based decision-making. Consequently, digital health offers significant benefits, such as enhanced patient engagement and adherence, while providing health care providers with data-driven insights for more precise diagnoses and treatment recommendations [5,6]. Beyond the mere incorporation of gadgets and software, digital health represents a profound shift in health care delivery, extending into daily lives through wearables, health apps, and web-based consultations [7]. This approach allows patients to conveniently access medical advice, monitor their health conditions, and manage chronic illnesses from their homes. At the same time, health care providers gain access to a wealth of data, potentially reducing errors and enhancing patient outcomes [8].

However, digital health also introduces new challenges, including data security, privacy concerns, system interoperability, regulatory compliance issues, and the need for fair access to health care technology. These issues are paramount in the health care industry [9]. Moreover, health care organizations and stakeholders need a structured framework to evaluate their maturity and ensure the effective adoption and integration of these technologies. The rapid pace of technological innovation has led to a fragmented landscape, with various digital health solutions operating on disparate standards and protocols [10]. The key challenge lies in facilitating seamless communication and data exchange between these systems while safeguarding patient privacy, representing no small feat.

To overcome these challenges, health care organizations aim to enhance their preparedness in the rapidly evolving digital health space. One path forward is to improve maturity. Maturity encompasses technological capabilities and the organization’s readiness to adapt and communicate effectively in this digital era [10]. This is where the digital health maturity model (DHMM) concept becomes significant [11]. Maturity models are currently used in many sectors, such as financial services and manufacturing [12]. These models offer a structured framework for assessing an organization’s readiness, progress, and effectiveness in adopting digital health solutions through the stages of maturation to optimize implementation and outcomes.

Typically, DHMMs consist of stages or levels that assess an organization’s capabilities in key areas such as technological infrastructure, data management, interoperability, clinical workflows, and patient engagement [10]. They help stakeholders identify gaps, prioritize needs, and determine a strategic and effective course for digital health implementation. However, existing DHMMs often fail due to technological, organizational, and social factors [12-14]. This results in a lack of a standardized DHMM for use in academia and public health practice.

### Objectives

To explore DHMMs more deeply, this review seeks to answer the following questions:

1. Which DHMMs have been used in research?
2. What are the common components among these models?
3. What technological, organizational, and social factors are insufficiently addressed by existing DHMMs?

On the basis of the findings, this study aims to propose a more comprehensive DHMM that addresses the limitations of current models and captures the dynamic nature of digital health.

## Methods

### Systematic Review

A systematic literature review was conducted based on the methodological frameworks of Arksey and O’Malley [15] and the JBI [16] to examine the existing literature on DHMMs. The PRISMA (Preferred Reporting Items for Systematic Reviews and Meta-Analyses) checklist [17] was applied to maintain methodological rigor, reduce bias, and ensure reliability (Multimedia Appendix 1). Following the approach outlined by Moosapour et al [18], the review synthesizes existing knowledge, addressing challenges related to technological advancements and varying digital health standards. It examines current DHMMs; identifies gaps in technological, organizational, and social factors; and outlines a framework for use in public health practice and academic contexts [18].

### Search Strategy

To identify relevant articles covering DHMMs, an extensive search strategy was used across EBSCO, PubMed, and ProQuest databases using the search term “Digital Health and Maturity” for publications from January 2000 to May 2024 (Multimedia Appendix 2 [19-49]). These databases were selected due to their established status in fields related to digital health [50]. Their prominence stems from several factors, including their extensive coverage of the literature, sophisticated cataloging systems, and user-friendly interfaces. Consequently, they are widely recognized and used by researchers, clinicians, and practitioners in digital health–related fields [51]. Furthermore, these databases offer advanced search options, access to full-text articles, and seamless integration with citation management tools, enhancing their utility when conducting focused reviews and meta-analyses in digital health research. Their credibility is bolstered by their long-standing presence in the academic and health communities, as well as their partnerships with reputable publishers, organizations, and networks [52]. Textbox 1 outlines the search strategy and terms used to identify relevant studies on DHMMs.

**Overview of the search strategy used to identify relevant digital health maturity models in this systematic review.**

**Steps and research terms**

1. (“Digital Health AND Maturity” [Title/Abstract])
2. (“Maturity Model” [Title/Abstract] OR “Maturity Index” [Title/Abstract] OR “Maturity assessment” [Title/Abstract] OR “Model in Digital Health” [Title/Abstract])

### Inclusion and Exclusion Criteria

The articles were reviewed and assessed based on their relevance to digital maturity in the development of a comprehensive framework tailored to the digital health landscape. In addition, only articles published in English were selected. Specific exclusion criteria were also used to refine article selection. The research excluded those articles that (1) did not contemplate maturity levels or categories, (2) were not related to the objectives of this study, (3) studied biological maturity, (4) focused on only IT implementation, and (5) were not full-text articles. In cases where multiple versions of a report were available, only the most recent version was included (Textbox 2).

**Inclusion and exclusion criteria used in this systematic review to identify relevant digital health maturity models from the selected databases.**

**Inclusion criteria**

- Article type: peer-reviewed journal articles, systematic reviews, and theoretical models
- Language: English
- Publication year: January 2000 to May 2024
- Keywords: must include terms such as “digital health,” “maturity model,” and “maturity index”
- Study population: studies on health care technologies or digital health
- Study design: observational, experimental, or review studies
- Outcome: studies focusing on digital health and communication models

**Exclusion criteria**

- Article type: conference abstracts, editorials, and non–peer-reviewed articles
- Language: non-English
- Publication year: articles published before 2000
- Keywords: articles lacking relevance to digital health or maturity-related terms
- Study population: studies unrelated to health or technology
- Study design: case reports or anecdotal evidence
- Outcome: studies unrelated to digital health and communication models

### Data Extraction

During the screening stages, articles were categorized into 2 dimensions. First, reference information, such as study title, publication year, journal, and authorship, was recorded. Second, each publication was evaluated for its relevance to the study’s objectives, methodology, outcomes, and recommendations regarding inclusion or exclusion. Each paper was thoroughly reviewed during the analysis to ensure alignment with this investigation’s objectives. The search results were then meticulously analyzed and reviewed using Microsoft Excel for further assessment. Rigorous data collection techniques were used, with trained personnel conducting data extraction. Any discrepancies that arose were resolved through discussions.

## Results

### Overview

The results section presents a systematic analysis of DHMMs, evaluating their key components, effectiveness, and limitations. The findings highlight gaps in technological, organizational, and social dimensions, and propose a new integrated model.

### Study Selection

**Figure 1:**
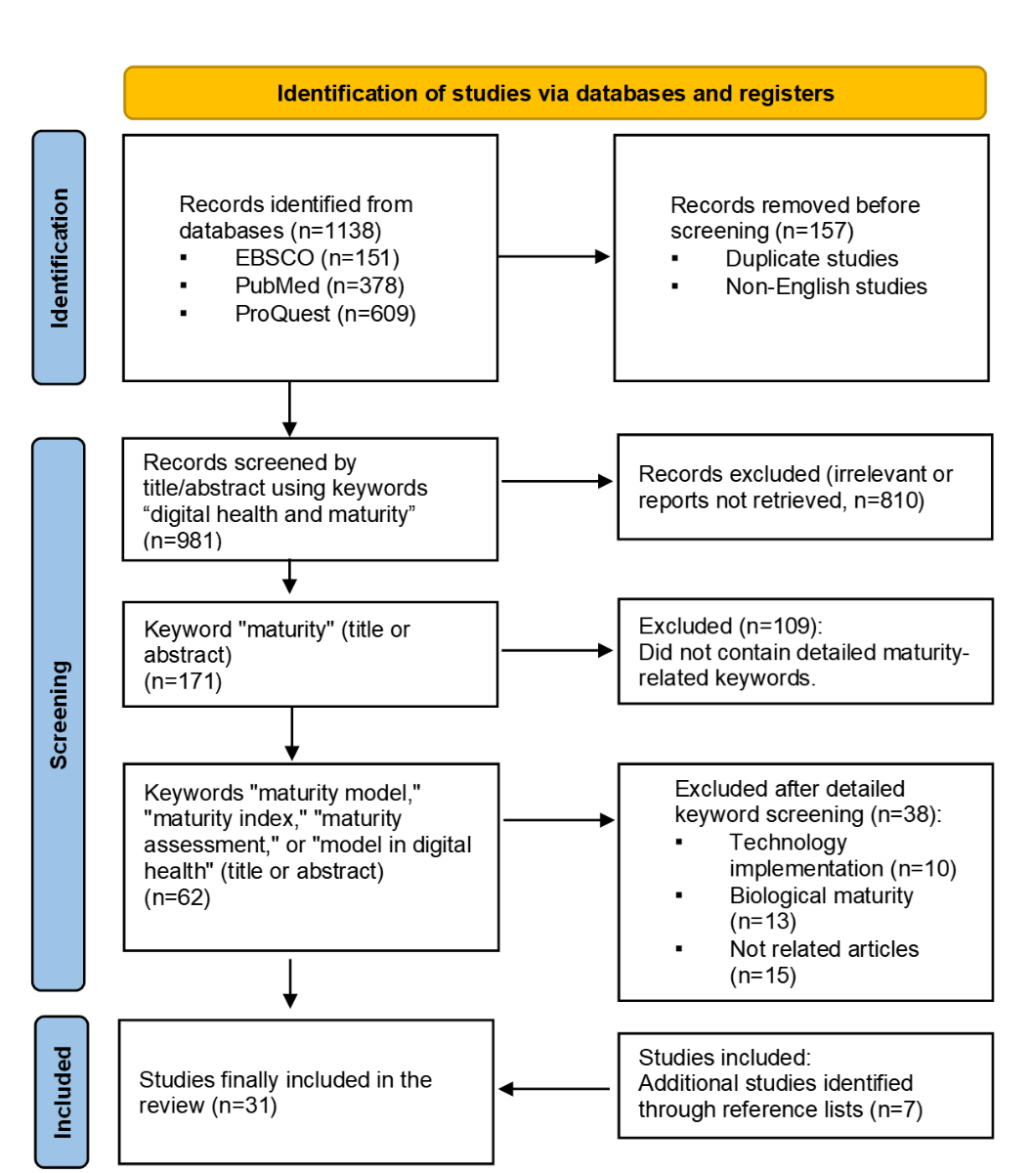
PRISMA (Preferred Reporting Items for Systematic Reviews and Meta-Analyses) flow diagram of the study selection process. The databases searched included EBSCO, PubMed, and ProQuest, using the search term “Digital Health and Maturity” for the period from January 2000 to May 2024. The process involved identification, screening, and inclusion phases. Inclusion criteria emphasized relevance to digital health maturity, health care frameworks, and full-text availability in the English language, while exclusion criteria ruled out non–health care studies, lack of empirical data, and non-English publications. The final selection included 31 studies for qualitative synthesis.

Initial searches yielded 1138 articles that were “relevant,” of which after excluding 157 (13.8%) duplicate and non-English articles, 981 (86.2%) remained for further evaluation. Of these 981 articles, 810 (82.6%) were excluded based on the absence of the key term “maturity” in their titles or abstracts, leaving 171 (17.4%) for continued assessment. Further refinement was applied to include only articles featuring terms such as “maturity model,” “maturity index,” “maturity assessment,” or “model in digital health,” resulting in a final selection of 62 articles, of which 15 (24%) unrelated studies, 13 (21%) studies on biological maturity, and 10 (16%) studies on implementation were excluded, leaving 24 (39%) studies eligible for inclusion in the review. The screening process is illustrated in the PRISMA flowchart (Figure 1). In total, 7 studies identified from the reference lists were also included, bringing the total number of studies for review to 31 (Table 1).

**Table 1:** The characteristics and key findings of studies on digital health maturity models (DHMMs) included in the systematic review.

Study	Methods	Source
Williams et al [19], 2019	Operational framework	*JMIR Medical Informatics*
Martin et al [20], 2019	Multivariable regression model	*NPJ Digital Medicine*
Gomes and Romão [21], 2018	State-of-the-art review	*Journal of Medical Systems*
Shaygan and Daim [22], 2019	Exploratory multicriteria approach	*IEEE Xplore*
van Dyk and Schutte [23], 2013	Iterative process	*Intech Open*
van Dyk and Schutte [24], 2011	Iterative process	*South African Journal of Industrial Engineering*
Jami Pour and Jafari [25], 2019	Mixed methods	*Emerald Insight*
Alexander et al [26], 2022	National survey	*JMIR Aging*
Duncan et al [27], 2013	Used various artifacts	*Online Journal of Public Health Informatics*
Duncan et al [28], 2022	Systematic review	*Journal of Medical Internet Research*
Eysenbach [29], 2007	Theoretical	*Studies in Health Technology and Informatics*
Grasser et al [30], 2018	Innovative, multidimensional, and human-centered socioeconomic capability model	*Studies in Health Technology and Informatics*
Jansen-Kosterink et al [31], 2022	Technology-readiness levels	*Digital Health*
Johnston [32], 2017	Digital maturity assessments	*Journal of Medical Internet Research*
Kayser et al [33], 2022	Narrative overview identifying health technology assessment–inspired models	*Informatics*
Kouroubali et al [34], 2019	Interoperability maturity model	*Studies in Health Technology and Informatics*
Kutza et al [35], 2022	Literature review and derivation of maturity objects	*Studies in Health Technology and Informatics*
Liaw et al [36], 2017	Mixed methods evaluation	*International Journal of Medical Informatics*
Liaw et al [37], 2021	Literature review	*Journal of the American Medical Informatics Association*
Nyangena et al [38], 2021	Health information system interoperability maturity toolkit, developed by MEASURE Evaluation and the Health Data Collaborative’s digital health and interoperability working group	*BMJ Health & Care Informatics*
Occelli and Scelfo [39], 2020	Survey	*International Journal of E-Planning Research*
Peytremann-Bridevaux et al [40], 2021	Scaling Integrated Care in Context maturity model tool	*BMJ Open*
Pumplun et al [41], 2021	Qualitative interview study	*Journal of Medical Internet Research*
Reifegerste et al [42], 2022	Telemedicine community readiness model	*Journal of Public Health*
Richmond et al [43], 2022	Novel maturity index for assessing medical device start-ups	*Journal of Clinical and Translational Science*
Stahl et al [44], 2022	Questionnaire study	*JMIR Formative Research*
Kolukısa Tarhan et al [45], 2020	Multivocal literature review	*Digital Health*
Tom-Aba et al [46], 2020	Evaluation of surveillance outbreak response management and analysis system using the global good maturity model version 1.0 indicators	*JMIR Public Health and Surveillance*
Flott et al [47], 2016	Systematic review	*Journal of Medical Internet Research*
Vogel et al [48], 2024	Used digital maturity data from the German DigitalRadar project (2021) and financial statement data from the Hospital Rating Report (2017-2019; n=860); performed linear regressions with the DigitalRadar score	*Health Policy*
Vogel et al [49], 2024	Performed multivariate linear regressions with the respective quality indicators as dependent variables and various digitalization subdimensions as independent variables	*Ruhr Economic Papers*

### Quality Evaluation

The selected articles were evaluated based on the quality of the scholarly publication using the Scimago Journal Rank indicator as the primary tool. This approach aligns with the one adopted by Oshni Alvandi et al [53]. Furthermore, each included paper underwent 2 assessment rounds, with the majority (22/31, 71%) being reviewed by a different researcher to ensure additional quality assurance (Table 2).

**Table 2:** Quality assessment of the included journals and articles using the Scimago Journal Rank indicator.

Rankings	Journals (n=30), n (%)	Studies (n=31), n (%)
Quartile 1	18 (60)	18 (58)
Quartile 2	4 (13)	4 (13)
Quartile 3	3 (10)	4 (13)
Quartile 4	0 (0)	0 (0)
Not yet assigned a rank	1 (3)	1 (3)
Not ranked	4 (13)	4 (13)

### Review of the Existing Maturity Models

The systematic review identified 8 commonly used DHMMs: the capability maturity model (CMM), CMM integration (CMMI), electronic medical record adoption model (EMRAM), continuity of care maturity model (CCMM), clinical digital maturity index (CDMI), interoperability maturity model (IMM), telemedicine service maturity model (TMSMM), and DigitalRadar maturity model (DRMM). These models were compared to discern how they evaluate digital health maturity levels and identify gaps and improvement opportunities. Combining various features from these models resulted in a new and more comprehensive model capable of providing detailed insights into an organization’s maturity level and highlighting avenues for growth. Detailed explanations of each of the models are provided in the following subsections.

### Detailed Explanations of DHMMs

#### CMM Framework

The CMM is a software engineering framework that outlines a 5-level path for systematically and incrementally improving software engineering processes. It was developed by the Software Engineering Institute at Carnegie Mellon University in the 1980s. It is widely used to assess the maturity of an organization’s software processes [53]. The model helps organizations identify and prioritize improvement areas based on their current level of process maturity. Organizations have few defined processes at level 1 (ie, the lowest level of process maturity). By contrast, at level 5 (ie, the highest level of process maturity), organizations have well-defined, highly optimized processes that are continually improved. The CMM focuses on process improvement and process maturity domains and subdomains, making it a valuable tool for organizations seeking to improve their software engineering processes [54,55] (Textbox 3 [53]).

**An overview of the 5 maturity levels of the capability maturity model, which represent an organization’s process maturity.**

**Levels and detailed descriptions**

- Level 1 (*initial*): the *initial* level is characterized by ad hoc and individualized processes. There is little or no process documentation, and processes are often not repeatable. At this level, the organization does not provide a stable environment for its software development activities.
- Level 2 (*repeatable*): the use of basic project management techniques characterizes the *repeatable* level. The organization begins to develop a set of repeatable standard processes from one project to the next and establish a project management infrastructure.
- Level 3 (*defined*): the use of formalized and standardized processes characterizes the *defined* level. All processes are well documented and approved by management. The organization has a clear and concise definition of the process used in all projects.
- Level 4 (*managed*): the active management of processes characterizes the *managed* level. The organization monitors and controls its processes using quantitative methods. Processes are continually improved and routinely audited.
- Level 5 (*optimized*): the proactive management of processes characterizes the *optimized* level. The organization continually strives to improve its processes based on a quantitative understanding and is constantly looking for ways to improve the efficiency and effectiveness of its software development processes.

#### CMMI Framework

The CMMI is a framework devised by the Software Engineering Institute at Carnegie Mellon University in the early 2000s. It offers a structured approach to enhancing software engineering processes [56]. It outlines a 5-level path for enhancing the processes incrementally (Textbox 4 [56]). The first 3 levels correspond to the capability levels of the CMM, and the fourth and fifth levels represent advanced improvement [57]. The CMMI has 4 main domains: *process*, *project*, *engineering*, and *support*. Each domain is further divided into subdomains [58]. The *process* domain includes planning, resourcing, deploying, implementing, monitoring, controlling, apprising, measuring, and improving processes.

**Overview of the 5 maturity levels of the capability maturity model integration, which reflect an organization’s process improvement and performance optimization capabilities.**

**Levels and detailed descriptions**

- Level 1 (*initial*): this level is the starting point for an organization’s journey toward process improvement. At this level, processes are typically ad hoc and chaotic, with little management.
- Level 2 (*managed*): the processes at this level are beginning to be more controlled and managed. There is now some standardization and documentation of processes, and teams have started to track and monitor their work more systematically.
- Level 3 (*defined*): the use of formalized and standardized processes characterizes the *defined* level. All processes are well documented and approved by management. The organization has a clear and concise definition of the processes used in all projects.
- Level 4 (*quantitatively managed*): the processes are not only well defined and standardized, but they are also quantitatively managed. This means that teams are monitored and controlled to ensure that they follow the defined processes and that performances are measured and analyzed to identify areas for improvement.
- Level 5 (*optimized*): the processes are continuously improved. This means that performances are measured and analyzed to identify areas for improvement, with changes then made to the processes to improve them.

The *project* domain encompasses project design, development, and validation, while the *engineering* domain covers system and software engineering activities, including development and maintenance. Finally, the *support* domain focuses on support product development and maintenance [57]. The CMMI framework helps organizations improve their processes and expand their capabilities.

#### EMRAM Framework

The EMRAM, created by the Healthcare Information and Management Systems Society (HIMSS), offers a comprehensive framework for evaluating the maturity of electronic medical record (EMR) systems within health care institutions [19]. Comprising 8 stages ranging from *preadoption* to *EMR optimization*, the model delineates the evolution of EMR adoption and use (Textbox 5 [59]). Developed in collaboration with clinicians and health care IT experts worldwide, the EMRAM integrates seamlessly into clinical workflows. It facilitates comparative assessments of EMR progress across organizations. Consequently, it serves as a vital tool for health care entities to gauge and enhance their EMR implementation, ultimately elevating the standard of patient care.

**An overview of the 7 maturity stages of the electronic medical record adoption model, a framework developed by the Healthcare Information and Management Systems Society to evaluate the adoption and implementation of electronic medical record (EMR) systems in health care organizations.**

**Stages and detailed descriptions**

- Stage 0 (*no EMR system in place*): this represents a stage where no EMR system is in place. This is the baseline level; all other levels are judged against it.
- Stage 1 (*EMR adoption—incomplete*): at this stage, the organization has started implementing EMRs but has not yet reached full adoption. The organization may be in the process of selecting an EMR system or may have already implemented an EMR system but is still in the process of training staff and integrating the EMR into the clinical workflow.
- Stage 2 (*EMR adoption—partial*): the organization has fully adopted EMRs in the clinical workflow at this stage. All staff members are trained on how to use the EMR system, and they use it daily to support patient care. The EMR is integrated into all aspects of the organization’s clinical workflow.
- Stage 3 (*EMR adoption—complete*): the EMR is used to track patient data and trends over time and is used to support evidence-based decision-making. All clinicians use the system for patient data storage, clinical decision support, electronic prescribing, and other features.
- Stage 4 (*EMR optimization—incomplete*): at this stage, EMRs are being used to improve patient safety. The EMR tracks patient safety data and trends and supports efforts to improve patient safety; for example, the system may be used for patient data storage, clinical decision support, electronic prescribing, and other features. However, there may be duplicate data entry issues, or the user interface could be improved.
- Stage 5 (*EMR optimization—partial*): at this stage, the EMR system is in place and being used to its full potential. Some improvements have been made in areas identified as needing improvement; for example, the system may be used for patient data storage, clinical decision support, electronic prescribing, and other features. The user interface may have been improved, or duplicate data entry may have been eliminated.
- Stage 6 (*EMR optimization—complete*): at this stage, EMRs are being used to transform health care. The EMR supports efforts to transform health care delivery through data-driven quality improvement initiatives.
- Stage 7 (*complete EMR; external health information exchange; and data analytics, governance, disaster recovery, privacy, and security*): organizations at this stage assess their experience with EMRs and make plans for their continued use or expansion. EMR systems are used for clinical care, with full functionality, integration across the care continuum, and evidence-based decision support.

#### CCMM Framework

The CCMM is a powerful framework that helps health care organizations deliver seamless patient care across multiple care sites and health care providers [21]. Developed by the HIMSS, the CCMM builds on the EMRAM, enhancing patient care by ensuring effective communication and teamwork among health care professionals. The CCMM delegates responsibility for coordinated care to administrators and governance leadership, clinical leadership, and IT and technology leadership, enhancing interoperability, governance, workforce efficiency, and predictive analytics within digital health [10].

The CCMM comprises the 5 domains of *governance*, *engagement*, *processes*, *information*, and *technology*, each with several subdomains that indicate the corresponding maturity level (Textbox 6 [59]). The *governance* domain includes organizational structure, policies and procedures, and risk management. The *engagement* domain covers values, beliefs, communication, training, and education. The *processes* domain focuses on care coordination, care transitions, and disease management. The *information* domain includes data collection, management, decision support, and reporting and analytics. Finally, the *technology* domain covers system architecture, security and privacy, and interoperability [60].

**An overview of the 7 maturity stages of the continuity of care maturity model, a framework for assessing the maturity of health care organizations in providing seamless and coordinated care across different health care settings.**

**Stages and detailed descriptions**

- Stage 0 (*limited or no electronic communication*): at this stage, there is little to no electronic communication between health care providers. This can make it challenging to coordinate care because health care providers may not have access to critical patient information.
- Stage 1 (*basic peer-to-peer data exchange*): at this stage, health care providers can exchange basic patient information electronically. This allows for better care coordination because health care providers can access vital patient information more easily.
- Stage 2 (*patient-centered clinical data exchanged using basic system-to-system exchanges*): at this stage, patient-centered clinical data are exchanged between health care providers using basic system-to-system exchanges.
- Stage 3 (*normalized patient record using structural interoperability*): at this stage, a normalized patient record is used to exchange patient information between health care providers, enabling better care coordination.
- Stage 4 (*care coordination based on actionable data using a semantic interoperable patient record*): at this stage, care coordination is based on actionable data using a semantically interoperable patient record where health care providers can access essential information.
- Stage 5 (*community-wide patient records using applied information with patient engagement focus*): community-wide patient records are used at this stage to exchange patient information between health care providers. This allows for better care coordination because health care providers can access important patient information more easily.
- Stage 6 (*closed-loop care coordination across care team members*): at this stage, closed-loop care coordination is used to exchange patient information for better care coordination.
- Stage 7 (*knowledge-driven engagement for a dynamic, multivendor, and multiorganizational interconnected health care delivery model*): at this stage, knowledge-driven engagement is used for dynamic, multivendor, and multiorganizational interconnected health care delivery, and health care providers access information.

Unlike the EMRAM, which focuses on specific aspects of EMRs, the CCMM takes a holistic approach to measuring maturity, emphasizing continuity of care and seamless patient information sharing among diverse health care providers. Developed collaboratively by the HIMSS and health care professionals worldwide, the CCMM is a valuable tool for health care organizations to assess, implement, and scale coordinated care.

#### CDMI Tool

The CDMI is a valuable tool introduced by the National Health Service in 2013. It was updated in 2016 to measure and track the progress of digital maturity within health care organizations in the United Kingdom [20]. It encompasses 3 key dimensions: *capability*, *technological readiness*, and *infrastructure*. The *capability* dimension assesses whether an organization possesses a well-defined digitalization strategy, effective governance structures, and adequately trained staff. The *technological* *readiness* dimension assesses the availability, effectiveness, and adaptability of technology to support digitalization efforts. Finally, the *infrastructure* dimension evaluates physical data and information management as well as the security infrastructure within the organization [20]. Each CDMI dimension is further divided into 14 sections, enabling a comprehensive assessment of an organization’s digital maturity level. With the CDMI, organizations can identify their strengths and weaknesses in terms of digital readiness and devise strategic improvement plans [60]. While the HIMSS maturity model may not be directly applicable in the UK context, the CDMI serves as a tailored and invaluable tool specifically designed for the UK market, offering a robust framework for assessing and advancing digital maturity within health care organizations. Furthermore, the framework facilitates monitoring progress and changes over time, fostering transparency and accountability (Textbox 7 [20]).

**An overview of the 7 maturity levels of the clinical digital maturity index, a tool designed to evaluate the maturity of clinical data management systems in health care organizations, with a focus on data collection, integration, and use for decision-making.**

**Levels and detailed descriptions**

- Level 0 (*digital maturity self-assessment*): organizations can assess their digital maturity using this tool. It is also possible to obtain independent assessments from an external organization to provide an objective view of an organization’s digital maturity and guidance on how to improve it.
- Level 1 (*themes*): various aspects of digital maturity are assessed, including readiness, capabilities, and infrastructure.
- Level 2.1 (*basic digital capabilities*): organizations typically have no formal processes for managing or improving their digital capabilities. They may have some digital capabilities, but these are not used effectively or efficiently.
- Level 2.2 (*developing digital capabilities*): organizations use digital technologies to improve patient care and clinical outcomes and develop digital capabilities.
- Level 2.3 (*advanced digital capabilities*): advanced digital technologies are used to improve patient care, and organizations adopt these technologies to transform how they work.
- Level 2.4 (*transforming digital capabilities*): organizations have formalized processes for transforming digital capabilities. They use data analytics to drive continuous improvements.
- Level 2.5 (*standardizing digital capabilities*): organizations are using digital technologies to standardize how they work and improve population health, which results in continuous improvements.

#### IMM Framework

The IMM was developed by the National E-Health Transition Authority of Australia to aid health care organizations in evaluating their level of interoperability maturity. Primarily, the IMM is structured around 5 stages of the CMMI, accompanied by a set of interoperability goals and an evaluation model geared toward the national level [21]. These stages are intricately linked to *organizational*, *informational*, and *technical* dimensions at various levels, including local, corporate, and national scales. The model delineates interoperability targets to enhance scalability, scope, and configurability across these dimensions [22].

The *organizational* dimension addresses business and governance considerations, ensuring alignment with strategic objectives. The *informational* dimension focuses on data format and semantics, ownership and rights, and standard building blocks, facilitating effective data exchange. Finally, the *technical* dimension encompasses specifications related to interface, functional decomposition, and communication protocols, which are vital for seamless interoperability. Although the IMM does not explicitly address architectural and technical policy separation, it furnishes health care organizations with a comprehensive framework to navigate their interoperability goals effectively [22]. By emphasizing these aspects, the IMM enables the development of robust interoperability strategies aligned with business objectives. Overall, it is a valuable tool for health care organizations to assess their interoperability maturity and devise plans for continuous improvement [52] (Textbox 8 [61]).

**An overview of the 5 maturity levels of the infrastructure maturity model, a framework designed to evaluate the maturity of an organization’s IT infrastructure in supporting digital transformation and operational efficiency.**

**Levels and detailed descriptions**

- Level 1 (*initial*): there is no interoperability.
- Level 2 (*managed*): the processes of this level are beginning to be more controlled and managed—a more customized approach aids in connecting technology to community specifications or standards.
- Level 3 (*defined*): interoperability at the data-sharing level is characterized by documenting and formalizing technologies in the community.
- Level 4 (*quantitively managed*): interoperability at this process level and sharing lessons with other communities do not require customization, given the existing pre-established standardization.
- Level 5 (*optimized*): purchasers of connected technology shall benefit from an improved interoperability performance language, and interoperability lessons will be implemented for continued improvements.

#### TMSMM Tool

The TMSMM is a comprehensive assessment tool designed to evaluate a telemedicine program’s readiness to implement and sustain a successful telemedicine initiative [23]. Developed by telemedicine practitioners from 5 South African provincial health departments, it focuses on various aspects of telemedicine application, particularly during the patient treatment stage [24]. This model encompasses 5 key characteristics—man, machine, material, method, and money—providing a holistic view of implementing a telemedicine service. The assessment is conducted through micro- and macro-level processes, with the maturity scale based on the generic-level indicators of the CMM.

However, the TMSMM consists of 5 levels, each representing a different stage of maturity in telemedicine service delivery. These stages are based on the program’s ability to provide telemedicine services effectively and efficiently and its readiness to expand and sustain these services over time. The 5 levels are the *initial stage*, the *experimental stage*, the *expansion stage*, the *consolidation stage*, and the *transformation stage*. At the *initial stage*, telemedicine services are in their infancy phase, with limited reach. Progressing to the *experimental stage*, services are tested and evaluated for effectiveness [62]. In the *expansion stage*, services are scaled up, extending their reach to more patients and health care providers. Upon entering the *consolidation stage*, telemedicine services are fully integrated into the health care system. They are becoming an integral part of the health care delivery model. Finally, the *transformation stage* represents the highest level of maturity, where telemedicine services have transformed the health care system and have led to significant improvements in patient outcomes, provider efficiency, and cost savings (Textbox 9 [24,25]).

**An overview of the 5 maturity levels of the telemedicine services maturity model, a tool for assessing the maturity of telemedicine services within health care organizations, focusing on the implementation, integration, and optimization of telehealth solutions.**

**Levels and detailed descriptions**

- Level 1 (*initial* [*ad hoc*]): telemedicine is used as needed, but no formal structure or governance exists.
- Level 2 (*managed*): telemedicine is used regularly, and some formal structures and governance are in place.
- Level 3 (*standard*): telemedicine is integrated into the health care delivery system, with strong stakeholder coordination.
- Level 4 (*quantitively managed*): telemedicine services have well-defined and managed processes in place. Processes are monitored, controlled, and continually improved.
- Level 5 (*optimized*): optimization is in place, and the telemedicine service is continuously monitored and improved. The goal is to achieve the highest level of efficiency and effectiveness possible.

#### DRMM Framework

The DRMM is a framework designed to assess digital transformation in hospitals, primarily focusing on health care institutions in Germany. It evaluates digital maturity by analyzing organizational processes, clinical applications, and technology infrastructure [63]. The model uses a structured web-based self-assessment through which hospitals evaluate their capabilities in clinical, administrative, and IT processes. These assessments generate individual scores, offer benchmarking insights, and guide strategies for digital transformation to improve patient care and hospital operations [63].

A key feature of the DRMM is its alignment with the funding requirements of Germany’s Hospital Future Act, linking digital maturity directly to external funding opportunities. This alignment incentivizes hospitals to engage in the self-assessment process. The model’s comprehensive methodology thoroughly evaluates IT performance, clinical workflows, system resilience, and telehealth capabilities [63].

The DRMM incorporates feedback from diverse stakeholders, including clinical staff, information management teams, and administrators, ensuring a broader representation of perspectives within the hospital’s digital ecosystem [48,63]. In addition, it provides a public dashboard for transparency, enabling hospitals to compare their scores against national benchmarks. This feature promotes competition and collaboration to improve health care delivery [48,63].

Despite its strengths, the DRMM has limitations. It is designed to closely align with Germany’s health care policies, which may restrict applicability in countries with different regulatory frameworks or digital infrastructures [63-65]. The reliance on self-reported data can introduce bias because hospitals might exaggerate their capabilities to meet funding criteria or performance targets [63]. Furthermore, while the model emphasizes technical and procedural dimensions, it gives limited attention to social and cultural factors, such as staff adoption of digital tools, which are crucial for effective digital transformation [63].

The DRMM outlines 7 maturity levels, describing the progression from basic infrastructure to fully integrated digital health systems. These levels are summarized in Textbox 10 [63], detailing critical aspects of digital health implementation [48,49,63].

**An overview of the 7 maturity levels of the DigitalRadar maturity model, a framework for assessing digital transformation progress in health care organizations by evaluating capabilities in digitalization, resilience, clinical processes, patient engagement, and interoperability.**

**Levels and detailed descriptions**

- Level 1 (*structures and systems*): focus on establishing the foundational IT infrastructure, including key performance indicators and software applications
- Level 2 (*resilience management and performance*): emphasis on IT security, resilience, and improving employee performance and satisfaction
- Level 3 (*organizational control and data management*): strengthening organizational control systems and enhancing data management processes
- Level 4 (*clinical processes*): integration of clinical processes, including decision support, order management, quality management, and flexible work processes
- Level 5 (*exchange of information*): implementing interoperability standards to facilitate information exchange between clinical staff, patients, and external entities
- Level 6 (*telehealth*): use of telemedicine technologies for remote consultations, emergency room connectivity, and telehealth networks
- Level 7 (*patient participation*): enabling patient access to information, participation opportunities, and use metrics to facilitate active patient engagement in their care

### Relationship of Maturity Models in Digital Health

**Figure 2:**
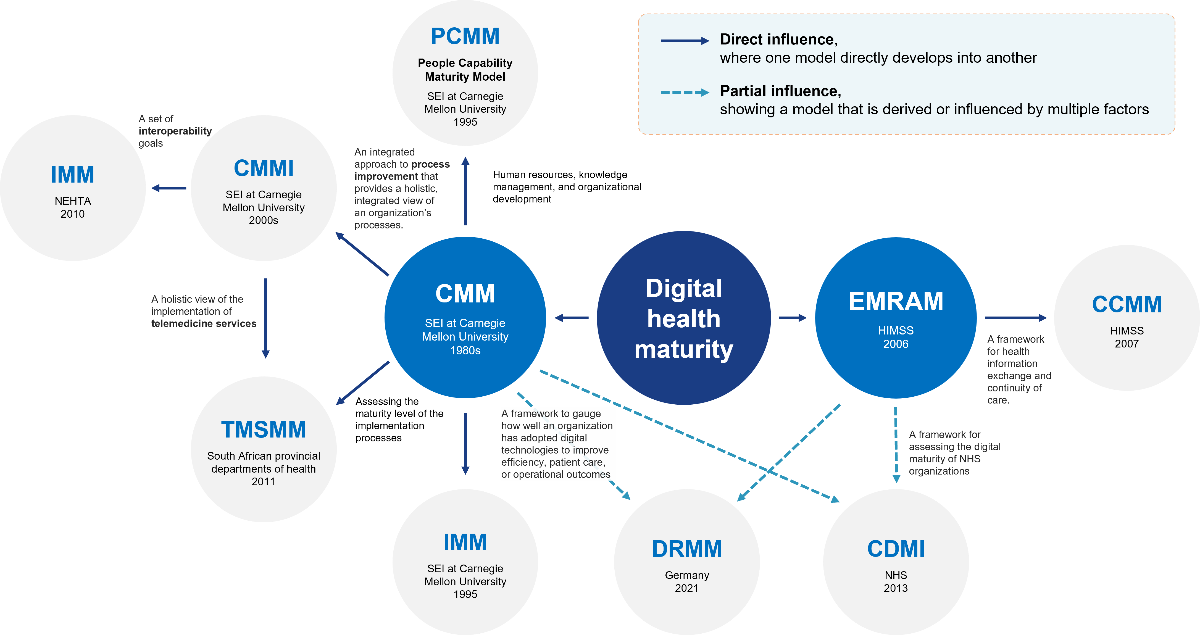
A comprehensive overview of the interrelationships among the 8 digital health maturity models, highlighting connections and interactions between frameworks, overlapping domains, complementary functionalities, and unique focus areas. CCMM: continuity of care maturity model; CDMI: clinical digital maturity index; CMM: capability maturity model; CMMI: capacity maturity model integration; DRMM: DigitalRadar maturity model; EMRAM: electronic medical record adoption model; HIMSS: Healthcare Information and Management Systems Society; IMM: interoperability maturity model; NEHTA: National E-Health Transition Authority of Australia; NHS: National Health Service; SEI: Software Engineering Institute; TMSMM: telemedicine service maturity model.

The review of maturity models has identified several frameworks, including CMMI, IMM, CDMI, TMSMM, and DRMM, which are influenced by the foundational CMM. These models adapt and expand CMM’s principles to address specific domains and needs within digital health. Meanwhile, the CCMM is influenced by EMRAM, as it extends the model’s focus on health information sharing among stakeholders to ensure continuity of care. These frameworks (CMMI, IMM, CDMI, TMSMM, and DRMM) collectively provide a structured approach to improving processes by aligning people, processes, and technology. The CMM serves as the conceptual foundation, while subsequent models enhance its scope to offer tailored strategies for digital health transformation. Figure 2 presents a detailed overview of the interrelationships among these DHMMs, illustrating how they build upon and complement each other to advance process improvement and digital health integration.

While the CMM was not initially designed for digital health, the EMRAM has emerged as one of the most widely adopted models due to its emphasis on assessing the maturity of EMRs, a critical component of digital health. The CCMM further extends this focus by facilitating health information sharing among stakeholders to ensure continuity of care. Similarly, the DRMM, which draws on the principles of the EMRAM, evaluates hospitals’ digital transformation by measuring progress in digitalization, system resilience, clinical processes, patient engagement, and information exchange.

Despite their widespread use, existing maturity models provide only a general framework for understanding the evolving digital health system. This broad focus on process improvement often limits their ability to fully capture the complexities and dynamic requirements of digital health system maturity [51]. As a result, these models may fall short of delivering the precise assessments needed for effective digital transformation in health care.

There is a pressing need for the refinement and further development of maturity models to ensure their continued relevance and efficacy. Enhanced models could provide a more comprehensive and detailed approach, better supporting the digital transformation efforts of health care systems.

### Analyzing the Maturity Levels and Domains of the 8 DHMMs

**Figure 3:**
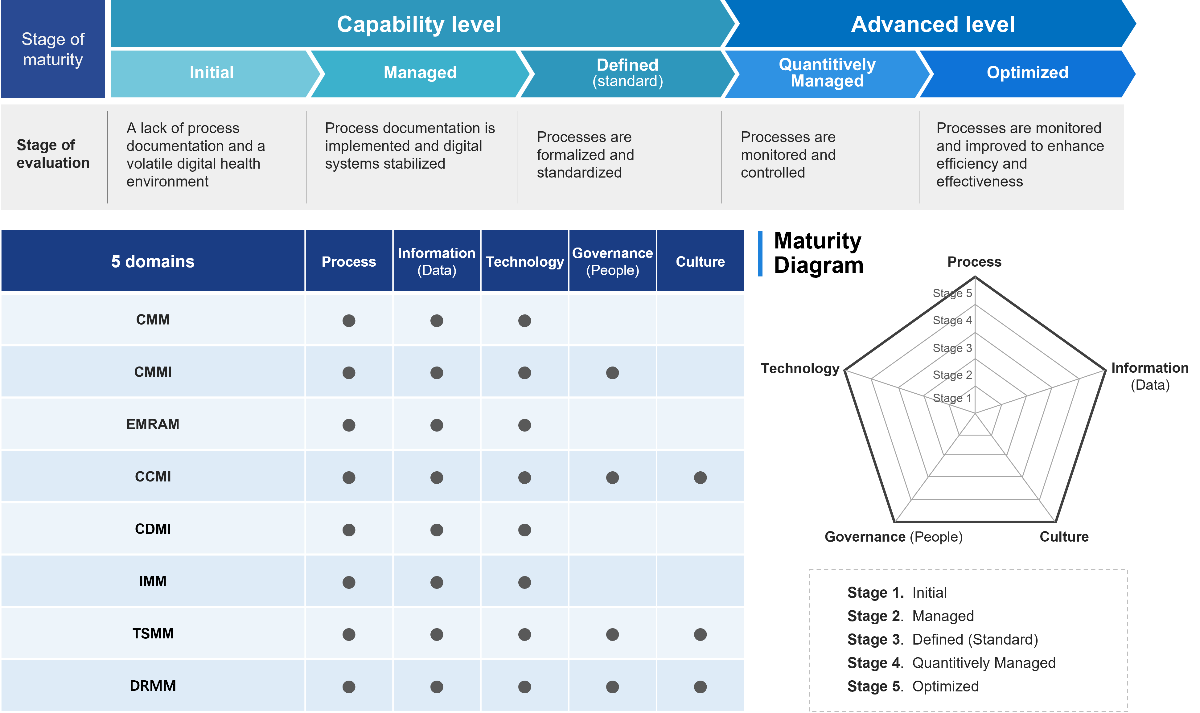
A comprehensive overview of existing digital health maturity models, categorized by their maturity levels and health care domains and illustrating their respective coverage and focus areas.

Table 3 presents an overview of the components and shared elements of the 8 maturity models, highlighting their differences and scope. The 5 maturity levels in these models are referred to here as *initial*, *managed*, *defined*, *qualitatively managed*, and *optimized* (Figure 3). The first 3 levels are capability stages focused on developing the capabilities of the digital health system. The remaining 2 levels are advanced stages focused on using basic capabilities. Each model’s domains can be separated into 5 categories: *process*, *information*, *technology*, *governance*, and *engagement*. These domains help to measure the organization’s execution of strategies, processes, and initiatives.

On the basis of a comprehensive review of this repository, insights into the detailed mechanisms of DHMM functionality are provided in response to the second research question. This involved aggregating information from diverse studies and models that rely on institutions such as the HIMSS and the World Health Organization as their sources, providing a clearer understanding of the key attributes of digital health maturity assessment. In addition, this review provides knowledge about how DHMMs have seemingly overlooked technologies, organizations, and societal issues. We also shed light on areas such as cybersecurity, governance structure development, the social determinants of health, and change management, which existing models overlook.

It is necessary to establish process documentation and digital systems stabilization at the *initial* level, followed by the *managed* level, which formalizes and standardizes the process. Subsequently, the *quantitatively managed* level enables the monitoring and control of standardized processes. Finally, the *optimized* level involves active monitoring and enhancing the process to maximize efficiency and effectiveness. The domains in each model have distinct categories. However, they can be separated into 5 categories, each critical for digital health maturity. Governance is essential to digital health maturity because collaborative agreements between stakeholders, legal frameworks, and regulations must be established as new processes emerge.

However, only half (4/8, 50%) of the existing models address the aspect of governance. The *engagement* domain components focus on the interaction between people and the degree of community involvement, with only 3 (38%) of the 8 models focusing on this domain. This research indicates that existing maturity models in digital health lack comprehensive coverage of critical aspects such as governance and engagement, which are essential for assessing maturity levels effectively. Therefore, developing a new integrated model is necessary to close these gaps and provide a more accurate digital health system maturity assessment.

**Table 3:** A summary of key characteristics, domains, and maturity levels of 8 digital health maturity models, including the capability maturity model (CMM), CMM integration (CMMI), electronic medical record adoption model (EMRAM), continuity of care maturity model (CCMM), clinical digital maturity index (CDMI), interoperability maturity model (IMM), telemedicine service maturity model (TMSMM), and DigitalRadar maturity model (DRMM).

Name of the model (developer; launch time frame)	Characteristics	Domains	Levels of process maturity
CMM (Software Engineering Institute at Carnegie Mellon University; 1980s)	A process improvement model that provides a framework for improving the quality of the software development process	Process improvement	Initial Repeatable Defined Managed Optimized
CMMI (Software Engineering Institute at Carnegie Mellon University; 2000s)	A process improvement approach that provides a framework for organizations to improve their processes	Process Project Engineering Support	Initial Managed Defined Quantitatively managed Optimized
EMRAM (Healthcare Information and Management Systems Society; 2006)	A framework for the implementation and use of EMRs^a^ in health care organizations	EMR adoption EMR use EMR data EMR workflow EMR decision support EMR reporting EMR optimization	No EMR system in place EMR adoption—incomplete EMR adoption—partial EMR adoption—complete EMR optimization—incomplete EMR optimization—partial EMR optimization—complete Complete EMR; external HIE^b^; and data analytics, governance, disaster recovery, privacy, and security
CCMM (Healthcare Information and Management Systems Society; 2007; updated in 2012 and 2017)	A framework for measuring the maturity of an organization’s continuity of care processes	Governance Engagement Processes Information Technology	Limited or no electronic communication Basic peer-to-peer data exchange Patient-centered clinical data using basic system-to-system exchange Normalized patient record using structural interoperability Care coordination based on actionable data using a semantic interoperable patient record Community-wide patient records using applied information with patient engagement focus Closed-loop care coordination Knowledge-driven engagement for dynamic, multivendor, and multiorganizational interconnected health care delivery model
CDMI (UK National Health Service; 2013; updated in 2016)	Measurement of the digital maturity of National Health Service organizations	Capability Technological readiness Infrastructure components	Digital maturity self-assessment Readiness, capabilities, and infrastructure Questions
IMM (National E-Health Transition Authority of Australia; 2010)	Measurement of interoperability associated with the technical, informational, and organizational capacities of the different players involved in health services	Data Process People Technology	Initial Managed Defined Quantitively managed Optimized
TMSMM (South African provincial departments of health; 2011)	A tool used to assess the readiness of a telemedicine program to implement and sustain a successful telemedicine initiative	Management Machine Material Method Money	Initial (ad hoc) Managed Standard Quantitatively managed Optimized
DRMM (Healthcare Information and Management Systems Society Europe and partners, including Institute for Applied Health Services Research; 2021)	A model for assessing digitalization in hospitals and evaluating funding impacts under the German Hospital Future Act	Evaluation of hospitals’ digitalization progress and resilience Clinical processes Patient participation Information exchange	Structures and systems Resilience Organizational control and data management Clinical processes Information exchange Telehealth Patient participation

^a^EMR: electronic medical record.

^b^HIE: health information exchange.

### Principal Results

#### Incorporating Communication Elements in DHMMs

Digital communication components within the DHMM framework are crucial in completing assessments. Unlike traditional DHMMs focused on technical features, communication dimensions practically include user connectivity, acceptance, personalization, and customization, which are critical factors in digital health platforms [51]. These elements can give DHMMs a coherent approach to evaluating a digital health management organization. They capture technical capabilities and examine the quality of patients’ and stakeholders’ interaction and communication experiences. This integration can uncover the benefits of digital health methods and progress regarding needs [20]. The process lays the groundwork for digital health projects to align with users’ needs and preferences, leading to more efficient and popular digital health solutions.

#### Introducing the Digital Health Communication Maturity Model

**Figure 4:**
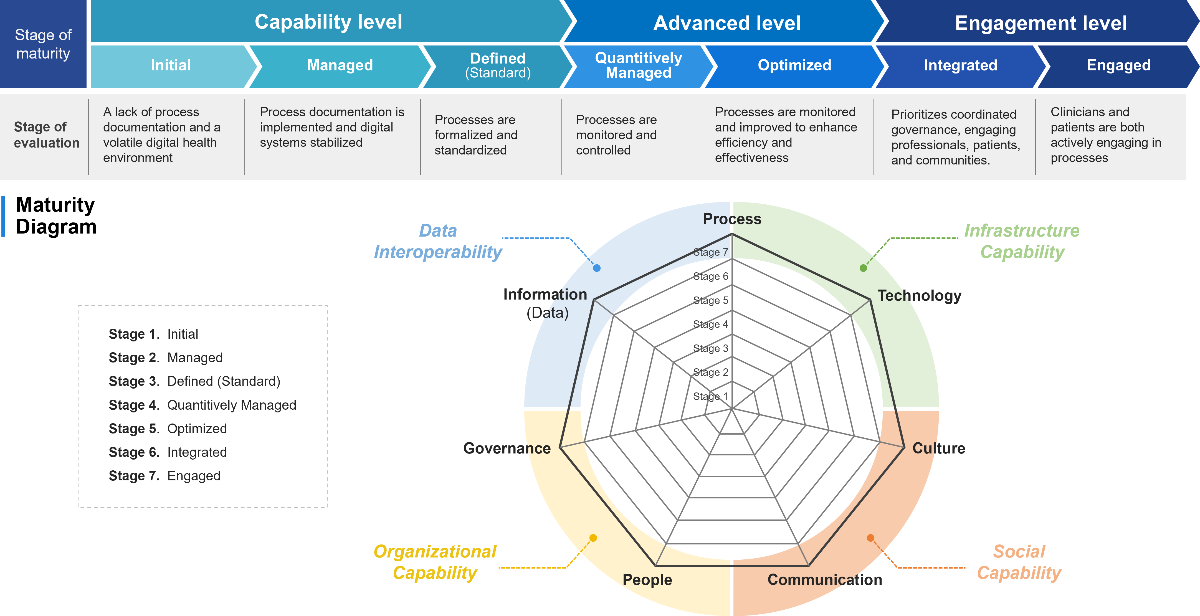
An overview of the digital health communication maturity model, a newly developed framework for evaluating digital health maturity with a distinct focus on communication dimensions often overlooked by other digital health maturity models.

The digital health communication maturity model (DHCMM) represents a paradigm shift in digital health maturity assessment. Unlike the other DHMMs, which tend to overlook the communication dimensions [25], the DHCMM is unique in that it incorporates specific user-centered metrics such as satisfaction, engagement, personalization, and customization into its framework (Figure 4).

Therefore, the DHCMM is ultimately key to assessing an organization’s readiness and capabilities for successfully implementing its digital health communication strategies [37]. By blending in communication models, the framework provides a more comprehensive take on the relationships among patients, digital health tools, and communication mediums, covering the aspects of channels, messaging, and user experience [38].

The core idea of the DHCMM is the dual requirement to have effective communication as a driver of technology and as the primary source of success in digital health initiatives [39]. Hence, communication strategies should be custom tailored to suit different user profiles, adapting to the evolving needs and preferences of a diverse population [40].

For a range of health care settings, from private clinics to public health campaigns and consumer-facing digital health care platforms, the DHCMM provides a structured approach to assessing communication effectiveness and identifying areas for improvement [20].

In other words, the DHCMM is a product of the latest digital technology development. Its introduction in digital health evaluation represents major progress because it offers a robust framework for assessing and improving communication practices in the digital age [39]. By instilling collaboration, governance, and meaningful engagements with eHealth applications, the model offers paths to more efficient and user-centered digital health development.

#### Enhancing Assessment and Optimization

Effective communication is crucial for the success of digital health initiatives, irrespective of the technological infrastructure [13]. At the same time, social factors such as user experience and communication significantly influence the acceptance and use of digital health solutions. Thus, overlooking these social capability aspects during digital health maturity assessments can undermine even the most advanced technological implementations [14]. In response to this dynamic nature of digital health, this review introduces the DHCMM, which addresses the limitations of existing DHMMs. The proposed model integrates DHMMs, communication, engagement factors, and technological dimensions, offering a precise road map and addressing crucial gaps to provide a nuanced view of an organization’s digital health maturity in the complex digital health landscape.

Current DHMM versions primarily focus on stabilizing the digital health system. However, it is imperative to incorporate bidirectional aspects such as the CCMM and DRMM to enhance user engagement at the community level. Such additions are expected to improve the operationalization of the digital health ecosystem, extending from hospital care to community care. In this context, an expanded maturity model, as depicted in Figure 4, introduces an *engaged* stage alongside the *capability* and *advanced* stages.

Level 6, the *integrated* level, emphasizes coordinated data and governance procedures, actively involving health care professionals, patients, and community members. Level 7, the *engaged* level, underscores the importance of the active involvement of organizations, community members, and physicians throughout the health care system.

A 7-level framework can be used to conceptualize digital health systems. The first 3 levels—*initial*, *managed*, and *defined* (*standard*)—constitute the *capability* stage. The *qualitatively managed* and *optimized* levels form the *advanced* stage. The *integrated* and *engaged* levels emphasize active user engagement and provide a platform for the active exchange of information while developing digital health capabilities in recently adopted areas and focusing on the effective use of advanced capabilities.

Overall, the proposed integrated DHCMM offers a more comprehensive approach to measuring the maturity of digital health systems, incorporating communication and engagement aspects not addressed by DHMMs. This model provides a framework for actively engaging users and stakeholders in the digital health ecosystem, emphasizing the importance of collaboration, governance, and the effective use of digital health capabilities.

#### The 7 Domains of Maturity for Optimal Outcomes

The DHCMM offers a comprehensive approach to evaluating digital health systems, incorporating crucial communication and engagement aspects overlooked by DHMMs. This model emphasizes collaboration, governance, and the effective use of digital health capabilities to actively engage users and stakeholders in the digital health ecosystem.

The 7 crucial domains of maturity—*process*, *information* (or *data*), *technology*, *governance*, *management*, *engagement*, and *communication*—collectively contribute to ensuring optimal outcomes in digital health systems. The *process* and *technology* domains focus on establishing the necessary infrastructure to support digital health initiatives. *Governance* and *management* are essential in efficiently applying digital health technology, encompassing organization-wide strategy, decision-making, and process management standards. Finally, the *engagement* and *communication* domains highlight the significance of social capabilities in successful digital health implementation. *Engagement* involves adapting to process changes, values, beliefs, attitudes, and behaviors, while *communication* emphasizes effective communication and cooperation within the process management framework.

The integrated DHCMM offers a comprehensive assessment of digital health system maturity, encompassing all relevant domains. Through its thorough coverage of these domains, the model ensures that systems are optimized for success, with a strong emphasis on user engagement and personalized care.

#### Evaluating Stakeholder Alignment

The effective execution of digital health programs hinges on collaborating with various stakeholders, including patients, legislators, health care professionals, and technology developers. While DHMMs have achieved significant progress in evaluating technology capabilities, the integrated DHCMM critically emphasizes stakeholder alignment. Efficient communication and cooperation among different groups are vital for aligning stakeholders and ensuring that the digital health ecosystem meets the requirements and expectations of all stakeholders. The DHCMM acknowledges the importance of this alignment within its *governance* and *engagement* domains. *Governance* establishes the legal frameworks and collaborative agreements, creating an environment conducive to digital health initiatives. It ensures that policies and regulations evolve alongside emerging technologies [13].

Moreover, the *engagement* domain underscores the importance of community involvement, user interaction, and effective communication techniques. Unlike traditional DHMMs focusing solely on technology infrastructure, the DHCMM acknowledges the broader scope of digital health maturity, considering the social aspects involved. It emphasizes active engagement from patients, health care professionals, and communities, recognizing their significant role in the success of digital health initiatives. The stakeholder-centered strategy of the DHCMM bridges the communication gap among important actors to achieve optimal outcomes. Likewise, technological proficiency calls for recognizing and addressing stakeholders’ various requirements, values, and beliefs [14]. This concept compels enterprises to consider the human factor in digital health, understanding that successful adoption requires more than cutting-edge technology.

#### Driving the Effective Use of Digital Health Capabilities

Furthermore, the integrated DHCMM expands on the topic beyond technology by emphasizing the role of stakeholder alignment in achieving digital health maturity. Through its assessment of the efficacy of communication, engagement, and governance frameworks, the model offers an all-inclusive road map for businesses to navigate the intricacies of the digital health terrain. While digital health technologies have revolutionized health care by improving communication and patient care, the lack of a standardized measure for digital health maturity presents a significant challenge. While existing maturity models are outlined in this review, a more detailed analysis is warranted.

The DHCMM also stresses the significance of persistent monitoring and evaluation in achieving progress in digital health communication strategies and battling misinformation. By latching onto key performance indicators and altering the approaches, organizations can tweak the use of their digital health capabilities and eventually improve the overall effect on health care outcomes.

## Discussion

### Principal Findings

This study explored the development of the DHCMM to address critical gaps in existing DHMMs. The DHCMM enhances traditional DHMMs by integrating communication, engagement, and governance as core metrics, bridging technical capabilities with user-centered dimensions. The findings support the study’s aim to propose a comprehensive model that captures the dynamic and multifaceted nature of digital health systems, emphasizing communication as a pivotal factor in achieving successful digital transformation. By incorporating stakeholder collaboration and advanced engagement strategies, the DHCMM goes beyond technical assessments to provide a framework for evaluating and advancing digital health maturity.

Traditional DHMMs, such as the CMMI and EMRAM, primarily prioritize technical metrics, including interoperability and data management [56,57,59]. However, their limited focus on user engagement and communication has restricted their applicability in addressing broader health care challenges. The inclusion of communication as a core domain within the DHCMM addresses this critical gap, aligning with research emphasizing the importance of patient-centered care and personalized health interventions [14,36].

The emphasis on communication aligns with the findings on mobile health of Smith and Magnani [14], which highlight the importance of enhancing digital health literacy and patient interaction to promote equitable access to digital health tools. Similarly, Alexander et al [26] underscored the structural disparities in health IT maturity, which can be mitigated by prioritizing communication and stakeholder alignment. By integrating engagement and governance, the DHCMM ensures a more holistic approach to digital health maturity, addressing the needs of diverse health care settings and populations. The DHCMM framework reflects the increasing demand for user-centered digital health solutions, aligning with the conclusions of Shaw et al [11] on the safety and quality benefits of digital interventions that incorporate patient portals and decision support systems. By emphasizing personalization and satisfaction, the DHCMM fosters improved adherence to digital health tools, as evidenced by Taj et al [66], who demonstrated the effectiveness of behavior change technologies when aligned with user preferences.

Moreover, the inclusion of governance and engagement in the DHCMM acknowledges the intricate relationship between technological capabilities and the social determinants of health. Nyangena et al [38] emphasized that governance structures and standardized technical frameworks are crucial for achieving effective interoperability and health information exchange. By incorporating governance as a core domain, the DHCMM builds upon these findings, enabling seamless collaboration among stakeholders while ensuring compliance with regulatory standards. The DHCMM’s emphasis on engagement addresses the challenges of user adoption and behavior change highlighted by Jansen-Kosterink et al [31]. Their work on continuous eHealth evaluation emphasizes the importance of iterative assessments that incorporate end-user, clinical, and societal perspectives [32]. By integrating these dimensions, the DHCMM offers a comprehensive road map for fostering sustainable digital health ecosystems.

The findings also reveal that existing DHMMs often lack specificity in evaluating communication and engagement, as noted by Duncan et al [28], who criticized current maturity assessments for being inadequate in hospital settings. The DHCMM’s 7-level framework, with stages such as *integrated* and *engaged,* provides a more refined evaluation that captures the dynamic evolution of digital health systems. This approach aligns with the work of Kolukısa Tarhan et al [45], who advocated for multicriteria maturity models to address the diverse and evolving needs of health care organizations.

While the DHCMM provides a comprehensive framework for assessing digital health maturity, several limitations should be considered. This model’s emphasis on communication and engagement may require significant cultural adaptation to align with diverse health care practices and societal norms. This aligns with the findings of Liaw et al [37], which highlighted the cognitive overload experienced by users when integrating complex digital health toolkits. Future iterations of the DHCMM should consider incorporating simplified pathways to mitigate these challenges and enhance usability. Furthermore, the model’s effectiveness in improving health outcomes depends on accurate and context-specific performance metrics. As Vogel et al [49] noted, existing quality indicators often lack the statistical sensitivity needed to measure the impact of digital transformation. Developing robust, outcome-oriented metrics will be crucial for validating the DHCMM’s efficacy.

The DHCMM represents a paradigm shift in digital health maturity assessment, emphasizing the critical role of communication, engagement, and governance in driving successful digital transformation. By integrating technical, social, and organizational dimensions, the model aligns with global efforts to enhance health care delivery through digital innovation.

### Conclusions

The broader implications of this study highlight the importance of collaborative, user-centered approaches to digital health. The DHCMM provides a versatile framework that overcomes the limitations of traditional DHMMs, fostering sustainable digital ecosystems that prioritize patient satisfaction, stakeholder alignment, and regulatory compliance. As health care systems navigate the complexities of digital transformation, the DHCMM serves as a road map for achieving maturity that is not only technologically advanced but also socially and ethically grounded. Future research should aim to validate the model’s applicability across diverse health care settings and refine its metrics to ensure continued relevance in the evolving digital health landscape.

## Data Availability

All data generated or analyzed during this study are included in Multimedia Appendix 2.

## Appendix

Multimedia Appendix 1 PRISMA checklist.

Multimedia Appendix 2 Database search strategy.

## Abbreviations

CCMM: continuity of care maturity model
CDMI: clinical digital maturity index
CMM: capability maturity model
CMMI: capacity maturity model integration
DHCMM: digital health communication maturity model
DHMM: digital health maturity model
DRMM: DigitalRadar maturity model
EMR: electronic medical record
EMRAM: electronic medical record adoption model
HIMSS: Healthcare Information and Management Systems Society
IMM: interoperability maturity model
PRISMA: Preferred Reporting Items for Systematic Reviews and Meta-Analyses
TMSMM: telemedicine service maturity model
