# Supplementary material for: Developing the Digital Health Communication Maturity Model: Systematic Review
Source: J Med Internet Res. 2025 Apr 14;27:e68344. doi: 10.2196/68344 (PMC12038289; doi:10.2196/68344)
Supplement: Multimedia Appendix 2 [file jmir_v27i1e68344_app2.docx]

1. **Databases search strategy I**

A thorough search approach was used across the PubMed, EBSCO and ProQuest databases from 2000 to May 2024 in order to find pertinent papers covering DHMMs. PubMed offers over 37 million citations from MEDLINE, life science journals, and books, linking to full-text resources from PubMed Central and publishers for biomedical research. EBSCO provides research databases, e-journals, e-books, and library technology for academic, corporate, and public libraries, supporting global institutions with clinical, educational, and management solutions. ProQuest delivers 90,000 authoritative sources, dissertations, newspapers, e-books, and historical collections, enabling advanced, multidimensional research and supporting libraries with SaaS solutions and bibliographic tools.

**Database limit:** The results of the research strategy were limited to the last 24 years (2000-2024)

**Review search strings used for each database**

| PubMed | ( "digital health"[tw] OR "digital healthcare"[tw] OR ehealth[tw] OR "e-health"[tw] OR mhealth[tw] OR "m-health"[tw] OR telehealth[tw] OR "tele-health"[tw] OR telemedicine[tw] OR "tele-medicine"[tw] OR "connected health"[tw] OR "smart health"[tw] OR "digital medicine"[tw] OR "electronic health"[tw] OR "mobile health"[tw] OR "virtual health"[tw] OR "digital transformation"[tw] OR "health information technology"[tw] OR "health it"[tw] OR "health technology"[tw] OR "digital therapy"[tw] OR "digital therapeutics"[tw] OR "digital mental health"[tw] OR "e-mental health"[tw] OR telepsychiatry[tw] OR "remote care"[tw] OR "remote monitoring"[tw] OR "remote patient monitoring"[tw] OR "digital hospital"[tw] OR "digital healthcare service"[tw] OR "digital care"[tw] OR "digital care model"[tw] OR "health 4.0"[tw] ) AND ( "maturity model"[tw] OR "capability maturity model"[tw] OR "maturity framework"[tw] OR "maturity matrix"[tw] OR "maturity approach"[tw] OR "maturity assessment"[tw] OR "maturity evaluation"[tw] OR "maturity taxonomy"[tw] OR "maturity level"[tw] OR "maturity index"[tw] OR "digital maturity"[tw] OR "digital maturity model"[tw] OR "capability maturity"[tw] OR "organizational maturity"[tw] OR "organizational maturity model"[tw] OR "organizational readiness"[tw] OR "readiness model"[tw] OR "readiness framework"[tw] OR "readiness approach"[tw] OR "readiness assessment"[tw] OR "readiness evaluation"[tw] OR "digital readiness"[tw] OR "digital transformation readiness"[tw] OR "healthcare readiness"[tw] OR "healthcare maturity"[tw] OR "healthcare maturity model"[tw] OR "CMM"[tw] OR "CMMI"[tw] ) AND (english[Filter]) |
| --- | --- |
| EBSCO | ((TI "digital health" OR AB "digital health") OR (TI ehealth OR AB ehealth) OR (TI telehealth OR AB telehealth) OR (TI "digital medicine" OR AB "digital medicine") OR (TI "mobile health" OR AB "mobile health")) AND ((TI "maturity model" OR AB "maturity model") OR (TI "digital maturity" OR AB "digital maturity") OR (TI "maturity assessment" OR AB "maturity assessment") OR (TI "organizational readiness" OR AB "organizational readiness") OR (TI "readiness model" OR AB "readiness model") OR (TI CMM OR AB CMM) OR (TI CMMI OR AB CMMI)) AND LA English |
| ProQuest | ( (ALL("digital health")) OR (ALL("digital healthcare")) OR (ALL(ehealth)) OR (ALL("e-health")) OR (ALL(mhealth)) OR (ALL("m-health")) OR (ALL(telehealth)) OR (ALL("tele-health")) OR (ALL(telemedicine)) OR (ALL("tele-medicine")) OR (ALL("remote care")) OR (ALL("remote monitoring")) OR (ALL("digital medicine")) OR (ALL("mobile health")) OR (ALL("virtual health")) OR (ALL("digital transformation")) OR (ALL("health information technology")) OR (ALL("health it")) OR (ALL("health technology")) OR (ALL("digital therapy")) OR (ALL("digital therapeutics")) OR (ALL("digital mental health")) OR (ALL("e-mental health")) OR (ALL(telepsychiatry)) OR (ALL("smart health")) OR (ALL("connected health")) OR (ALL("digital hospital")) ) AND ( (ALL("maturity model")) OR (ALL("capability maturity model")) OR (ALL("digital maturity")) OR (ALL("maturity assessment")) OR (ALL("maturity evaluation")) OR (ALL("maturity framework")) OR (ALL("maturity matrix")) OR (ALL("maturity approach")) OR (ALL("organizational readiness")) OR (ALL("readiness model")) OR (ALL("readiness approach")) OR (ALL("readiness assessment")) OR (ALL("readiness evaluation")) OR (ALL("digital readiness")) OR (ALL("digital transformation readiness")) OR (ALL(CMM)) OR (ALL(CMMI)) ) AND la.exact("English") |

**B.  Databases search strategy II**

| **Step** | **Description** | **PubMed** | **EBSCO** | **ProQuest** | **Total** |
| --- | --- | --- | --- | --- | --- |
| Raw Keyword Search | Articles identified through database searches. Keywords: Digital Health, Maturity, Maturity Models, etc. | 378 | 151 | 609 | 1138 |
| Duplicates and Non-English Removed | Removed duplicate records and non-English studies. | 54 | 32 | 71 | 157 |
| Identified for Digital Health | Articles identified focusing on digital health and maturity. | 324 | 119 | 538 | 981 |
| Reports Not Retrieved | Records screened but not retrieved. | 275 | 96 | 439 | 810 |
| Title/Abstract Screening | Articles screened for relevance based on title/abstract. | 49 | 23 | 99 | 171 |
| Focused Screening | Articles focusing on maturity models, maturity indices, assessments, or models in digital health. | 19 | 11 | 32 | 62 |
| Excluded Studies | Excluded articles based on criteria: Technology implementation (n=10), Biological maturity (n=13), Unrelated (n=15). | 11 | 7 | 20 | 38 |
| Studies Included | Studies selected for review from databases. | 8 | 4 | 12 | 24 |
| Additional Studies | Additional relevant studies identified from references. | - | - | - | **7** |
| Total Studies Included | Final number of studies included in the review. | 8 | 4 | 12 | **31** |

**C.  Databases Inclusion and Exclusion Criteria II**

| **Criteria** | **Inclusion Criteria** | **Exclusion Criteria** |
| --- | --- | --- |
| Article Type | Peer-reviewed journal articles, systematic reviews, theoretical models | Conference abstracts, editorials, non-peer-reviewed articles |
| Language | English | Non-English |
| Publication Year | 2000-2024 | Articles published before 2000 |
| Keywords | Includes terms like "Digital Health," "Maturity Model," "Maturity Index" | Articles lacking relevance to digital health or maturity-related terms |
| Study Population | Studies on healthcare technologies or digital health | Studies unrelated to health or technology |
| Study Design | Observational, experimental, or review studies | Case reports or anecdotal evidence |
| Outcome | Studies focusing on digital health and communication models | Studies unrelated to digital health and communication models |
